# Non‐Stoichiometric Amorphous Calcium Carbonate Forms in Macromolecular Condensates via Interphase Diffusion

**DOI:** 10.1002/smll.202411965

**Published:** 2025-01-16

**Authors:** Debojit Paul, Neta Varsano, Protap Biswas, Ifat Kaplan‐Ashiri, Lior Aram, Assaf Gal

**Affiliations:** ^1^ Dept. of Plant and Environmental Sciences Weizmann Institute of Science Rehovot 7610001 Israel; ^2^ Dept. of Chemical Research Support Weizmann Institute of Science Rehovot 7610001 Israel

**Keywords:** amorphous calcium carbonate, biomineralization, crystallization, liquid–liquid phase separation, polymer induced liquid precursor

## Abstract

Transient amorphous phases are known as functional precursors in the formation of crystalline materials, both in vivo and in vitro. A common route to regulate amorphous calcium carbonate (ACC) crystallization is via direct interactions with negatively charged macromolecules. However, a less explored phenomenon that can influence such systems is the electrostatically driven formation of Ca‐macromolecule dense phases. In this study, it is shown how Ca‐macromolecule condensates that form via liquid–liquid phase separation (LLPS) can be used to control the formation of metastable ACC via diffusion‐based mass transport. Contrary to the solid‐like ACC particles that form in the dilute phase via rapid nucleation and growth, the condensate ACC gradually forms via carbonate diffusion into the dense droplets. This yields transient phases with non‐stoichiometric compositions, similar to a solid solution. It is shown that the ability to control the concentration gradients across the phase boundary can be used to finely regulate the composition and stability of these amorphous precursors, offering new routes to control mineralization through transient phases.

## Graphical abstract

This study shows how polymer‐calcium condensates can be used as scaffolds for the formation of metastable amorphous calcium carbonate. The condensates are the result of liquid–liquid phase separation between the polymer and calcium solutions, with carbonate diffusion gradually changing their composition. This two‐stage process is driven by equilibrium dynamics, and thus can represent biomineral formation in vivo.

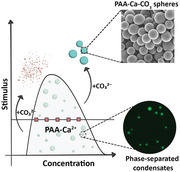

## 1 Introduction

Crystallization from aqueous solutions is intensively studied.^[^ ^1^, ^2^, ^3^, ^4^, ^5^ ^]^ It appears that the reductionist perspective of the classical nucleation theory needs to be much expanded to encompass the complexities of real‐world reactions. The calcium carbonate system is a model for studying various crystallization pathways as it is very common in geologic, biogenic, and synthetic settings. Over the years, the involvement of metastable transient phases as precursors to the final crystalline materials became a paradigm of the field.^[^ ^3^, ^6^, ^7^, ^8^, ^9^ ^]^ These precursors lack long‐range order and were termed amorphous calcium carbonate (ACC), but it is now clear that there are myriad types of ACCs that vary in their structure, stability, hydration level, and many other traits.^[^ ^10^ ^]^

The formation and transformation of ACC are usually described as phase transitions when a new chemical phase forms from a preceding condition. The most common phase transition in this regard is nucleation and growth, a process that is dependent on various factors such as cluster size and solution additives.^[^ ^2^, ^11^, ^12^, ^13^ ^]^ Other scenarios for ACC formation include an initial liquid–liquid phase separation (LLPS) that gives rise to a dense liquid phase, rich in inorganic building blocks.^[^ ^11^, ^14^, ^15^, ^16^, ^17^, ^18^, ^19^ ^]^ All these phase transitions are characterized by abrupt structural and compositional changes. However, usually, they maintain a 1:1 stoichiometric ratio between calcium and carbonate with a varying degree of hydration. This trait is needed for the conservation of charge neutrality, and in cases where other species, such as bicarbonate, are involved, the stoichiometry changes accordingly.^[^ ^14^ ^]^

Negatively charged macromolecules are known as potent regulators of the stability of various ACC phases.^[^ ^20^, ^21^, ^22^ ^]^ These polymers were shown to effectively stabilize ACC even at minute concentrations at the µm range due to their potent activity as nucleation inhibitors of the crystalline phases.^[^ ^23^, ^24^ ^]^ When more concentrated polymer solutions, in the mm range, are used, a liquid‐like form of ACC is formed.^[^ ^16^, ^25^, ^26^, ^27^ ^]^ This polymer‐induced liquid precursor (PILP) leads to a subsequent crystallization process that gives rise to biomimetic crystalline features.^[^ ^20^, ^28^, ^29^, ^30^ ^]^ Such PILP‐mediated processes are known to be advantageous for the formation of functional organic–inorganic hybrid materials. It is, therefore, important to understand how to control the PILP process.

Importantly, elevating the concentration of polymer functional groups to the hundreds of mm range will bring the system into a state where the polymer is even more concentrated than the inorganic ions. In this regime, electrostatic interactions between the negatively charged polymers and calcium cations dominate and can lead to complex coacervation.^[^ ^29^, ^31^, ^32^, ^33^ ^]^ Coacervation is one of few terms to describe a system where associative interactions between components lead to de‐mixing and the formation of two liquid phases through LLPS, one dilute in solutes and one dense.^[^ ^34^, ^35^ ^]^ Such macromolecular condensates are very common in crowded environments within living cells, and their physicochemical properties are known to regulate biochemical reactions.^[^ ^36^, ^37^, ^38^ ^]^ Even though biomolecular condensates have been intensively studied in recent years, their possible relation to biomineralization processes lags behind other cellular activities.

In this work, we explore the hypothesis that separating a PILP‐like process into two distinct steps: a first step, which includes condensation of polymer and Ca^2+^ ions through LLPS, and a second step where carbonate is introduced into the polymer‐Ca condensate, can be used to control the formation and composition of ACC. Our results show that carbonate diffuses into the polymer‐Ca condensates, yielding a supersaturated polymer‐CaCO_3_ phase that can crystallize. In this process, the compositional change that leads to ACC formation is driven by diffusion across an interface, whereas the structural change that leads to crystallization is driven by increasing metastability of the ACC. The decoupling between the two processes allows to finely regulate the composition and stability of the polymer‐CaCO_3_ phase.

## 2 Results and Discussion

**Figure 1:**
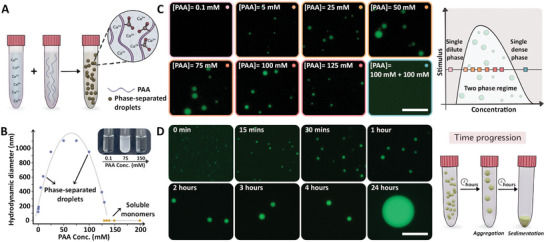
Liquid–liquid phase separation of PAA and Ca^2+^ solutions. A) A scheme of the components in our experimental system. B) Sizes of particles detected by DLS immediately after mixing of 50 mm Ca^2+^ and PAA solutions. The inset shows images of the concentration‐dependent turbidity. See Figure S1 (Suppporting Information) for raw DLS datasets. C) Fluorescence images of the PAA‐Ca phase‐separated droplets at 50 mm Ca^2+^ and various [PAA]. A schematic and color‐coded phase diagram shows the different regimes observed. D) Fluorescence images show time‐dependent growth of the dense droplets formed at 50 mm Ca^2+^ and 100 mm PAA. Scale bars are 20 µm and apply to all micrographs. See Figures S2 and S3 (Suppporting Information) for size analyses of the droplets.

To establish a Ca‐polymer coacervate system, various concentrations of 1200 kDa polyacrylic acid (PAA) solutions and a 50 mm Ca^2+^ solution, both titrated to pH = 9, were mixed (**Figure** **1**A). A clear indication of a phase separation process was the transformation of the solution from transparent to turbid immediately upon mixing (Figure 1B inset). Dynamic light scattering (DLS) was used to detect the sizes of the formed particles. The system showed particle formation above 0.2 mm PAA (Figures 1B, Figure S1, Suppporting Information, note that polymer concentrations refer to the concentration of the carboxylic acid residues). The size of the particles increased with PAA concentration, with a maximal hydrodynamic radius of ≈1.1 µm at a concentration of 50–75 mm. Beyond this concentration, the average size diminished. Above 130 mm, only nanometer‐sized particles were detected, which we attribute to soluble polymer molecules. This behavior is characteristic of associative LLPS (a schematic phase diagram is shown in Figure 1C).^[^ ^34^ ^]^

In order to image the forming particles, we added 0.75% fluorescently labeled 100 MDa PAA to the non‐labeled PAA solutions. In accordance with the DLS data, fluorescent droplets were observed in a concentration‐dependent manner (Figures 1C, Figure S2, Suppporting Information). We tested the liquid nature of the dense droplets by following a mixture of 100 mm PAA and 50 mm Ca^2+^ at different time points. The fluorescence images showed that the droplets grew with time, eventually sinking gravitationally (Figures 1D, Figure S3, Suppporting Information). This indicates droplet coalescence that is driven by minimization of the interfacial area that is facilitated by the liquid nature of the dense droplets.^[^ ^39^ ^]^ The LLPS behavior was further confirmed by adding PAA to a condition in the two‐phase regime, which shifted the system to a single dense phase regime and led to the dissolution of the droplets (Figure 1C last condition).

**Figure 2:**
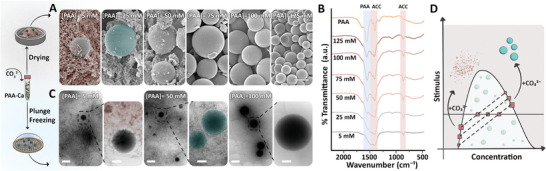
Adding carbonate to the phase‐separated PAA‐Ca system results in two distinct ACC phases. A) SEM images and B) FT‐IR spectra of dried PAA‐Ca‐CO_3_ samples synthesized with 50 mm Ca^2+^ and variable PAA concentrations. A powdered PAA sample is used as a control. The scale bar for all images is 1 µm. C) Cryo‐TEM images of samples from the same experiments in their hydrated state. The high‐magnification images are of similar spheres from the same vitrified grids as the low‐magnification images. Scale bars are 1 µm and 200 nm for the low and high‐magnification images, respectively. D) A scheme of the effect of carbonate addition to the phase‐separated system. In the unstable regime, the PAA‐Ca system separates to concentrated droplets and a dilute bulk. Adding carbonate leads to ACC nanoparticles forming in the bulk and the formation of PAA‐Ca‐CO_3_ spheres.

After exploring the LLPS behavior of the PAA‐Ca system we moved to the next step, adding a CO_3_^2‐^ solution. To account for the time‐dependent coalescence of the PAA‐Ca droplets, and have a constant size distribution of droplets across experiments, we decided to add 25 mm of CO_3_^2−^ at a constant time point of one hour after mixing the PAA and Ca solutions. The introduction of CO_3_^2‐^ immediately leads to additional visible turbidity in the solution, suggesting ACC precipitation. This additional turbidity was more pronounced at the low PAA concentrations (Figure S4, Suppporting Information). SEM imaging of dried PAA‐Ca‐CO_3_ mixtures showed two distinct morphologies, granular nanoparticles and micron‐sized spheres (**Figure** **2**A). The abundance of spheres significantly increased with increasing PAA concentration, being dominant above 75 mm. To verify that the micron‐sized spheres originate from the dense PAA‐Ca droplets, we confirmed that the size and abundance of fluorescent spheres before and after carbonate addition are similar (Figure S5, Suppporting Information). However, the PAA‐Ca droplets, which were initially liquid, solidified upon CO_3_^2−^ addition, as inferred from the observation that they did not coalesce with time (Figure S6, Suppporting Information).

We analyzed the dried and washed samples with FT‐IR to investigate their chemical composition. The spectra showed the characteristic ACC peaks, and a PAA peak with a magnitude that correlates with PAA concentration in the sample (Figure 2B). Similarly, thermogravimetric analyses (TGA) showed a higher PAA weight loss in samples prepared with elevated PAA (Figure S7, Suppporting Information). These measurements indicate that carbonate addition led to the formation of ACC‐polymer hybrid that consists of two morphological types (nanoparticles and microspheres), whose polymer content depends on the initial coacervation conditions. The different polymer content of nanoparticles and spheres is supported by a gravitational separation between the two precipitates, which is feasible at specific conditions that allow the nanoparticles to sink to the bottom faster than the spheres. When the two separated products were analyzed with TGA, the spheres showed 6% more weight loss due to the polymer compared to the nanoparticles (Figure S8, Suppporting Information).

To investigate if the drying procedure can skew the interpretation of the results, we imaged the samples in their native hydrated state using cryo‐TEM. Characterizing three different samples showed morphological observations in accordance with the SEM images (Figure 2C). Namely, the coexistence of nanoparticles and larger spheres, with a relative abundance related to the initial LLPS condition. The electron diffraction patterns of these samples confirm their amorphous phase (Figure S9, Suppporting Information). Altogether, these experiments show that the addition of carbonate to the phase‐separated PAA‐Ca system had a different effect on the dilute and dense PAA‐Ca phases (Figure 2D). In the dilute phase, carbonate addition triggers a second phase separation reaction, the precipitation of ACC nanoparticles. However, in the dense PAA‐Ca droplets, carbonate infiltrates these PAA‐Ca condensates, changing their chemistry into a new PAA‐Ca‐CO_3_ phase.

**Figure 3:**
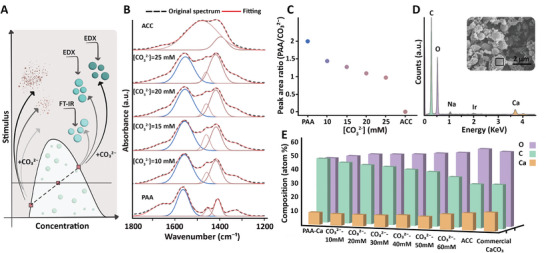
Formation and characterization of non‐stoichiometric PAA‐Ca‐CO_3_ spheres. A) Schematic showing the addition of variable [CO_3_^2−^] to the phase‐separated PAA‐Ca system and the analyses of the forming PAA‐Ca‐CO_3_ spheres. B) Gaussian peak fitting of the FT‐IR spectra collected from spheres synthesized with different [CO_3_^2−^], and the two pure end‐members of powdered PAA and inorganic ACC. The three main peaks (one PAA peak in blue and two ACC peaks in lilac) are shown. See Figure S12 (Suppporting Information) for a complete data fitting. C) Ratios between the PAA and carbonate FT‐IR peak areas in (B) demonstrate lower PAA content with increasing added carbonate. D) Site‐specific EDS spectrum collected from a sphere in a sample that contains both ACC nanoparticles and condensate spheres (the inset shows an example of the analyzed rectangular area of a single sphere). The spectra are not fully quantitative because of the low energies of the C and O edges and sample roughness. Nevertheless, a qualitative trend in the elemental compositions can be extracted. E) Analyses of EDS data from ACC spheres synthesized with different [CO_3_^2−^]. The trend shows a gradual transition from a PAA‐Ca composition to ACC.

From this point we turned to investigate the content of carbonate in the PAA‐Ca‐CO_3_ condensates and fixed the experimental condition of the PAA‐Ca system to 100 mm PAA and 50 mm Ca^2+^, with varying concentrations of CO_3_^2−^ (**Figure** **3**A). When only 10 to 25 mm CO_3_^2−^ are added to the solution no ACC nanoparticles form in the dilute phase and the dominant precipitates are the condensate spheres (Figure S10, Suppporting Information). This allowed to use FT‐IR spectroscopy of dried bulk samples as a reporter for the composition of the PAA‐Ca‐CO_3_ spheres. The spectra showed a clear trend of higher carbonate peaks as more carbonate was introduced (Figure 3B,C), implying a variable composition of the PAA‐Ca‐CO_3_ spheres. The observation of a non‐stoichiometric PAA‐Ca‐CO_3_ phase is further supported by TGA analyses showing higher weight loss due to polymer content for samples with lower [CO_3_^2−^] (Figure S11, Suppporting Information).

We wanted to quantify the compositional trend of the spheres when more than 30 mm CO_3_^2−^ was added and ACC nanoparticles formed in the dilute phase. To this end, we used site‐specific energy dispersive spectroscopy (EDS) (Figure 3D). We took advantage of the different C to O ratios in PAA‐Ca condensates and ACC, and measured the content of C, O, and Ca in PAA‐Ca‐CO_3_ spheres that were synthesized with varying concentrations of carbonate. This semi‐quantitative analysis of samples that contain up to 60 mm CO_3_^2−^ shows a trend in the C to O ratio toward the value of ACC (Figure 3E). Altogether, the spectroscopy analyses suggest that the PAA‐Ca droplets acquire a variable amount of carbonate from their environment. This leads to a non‐stoichiometric ratio of Ca and carbonate, in contrast to ACC formation via nucleation and growth.

**Figure 4:**
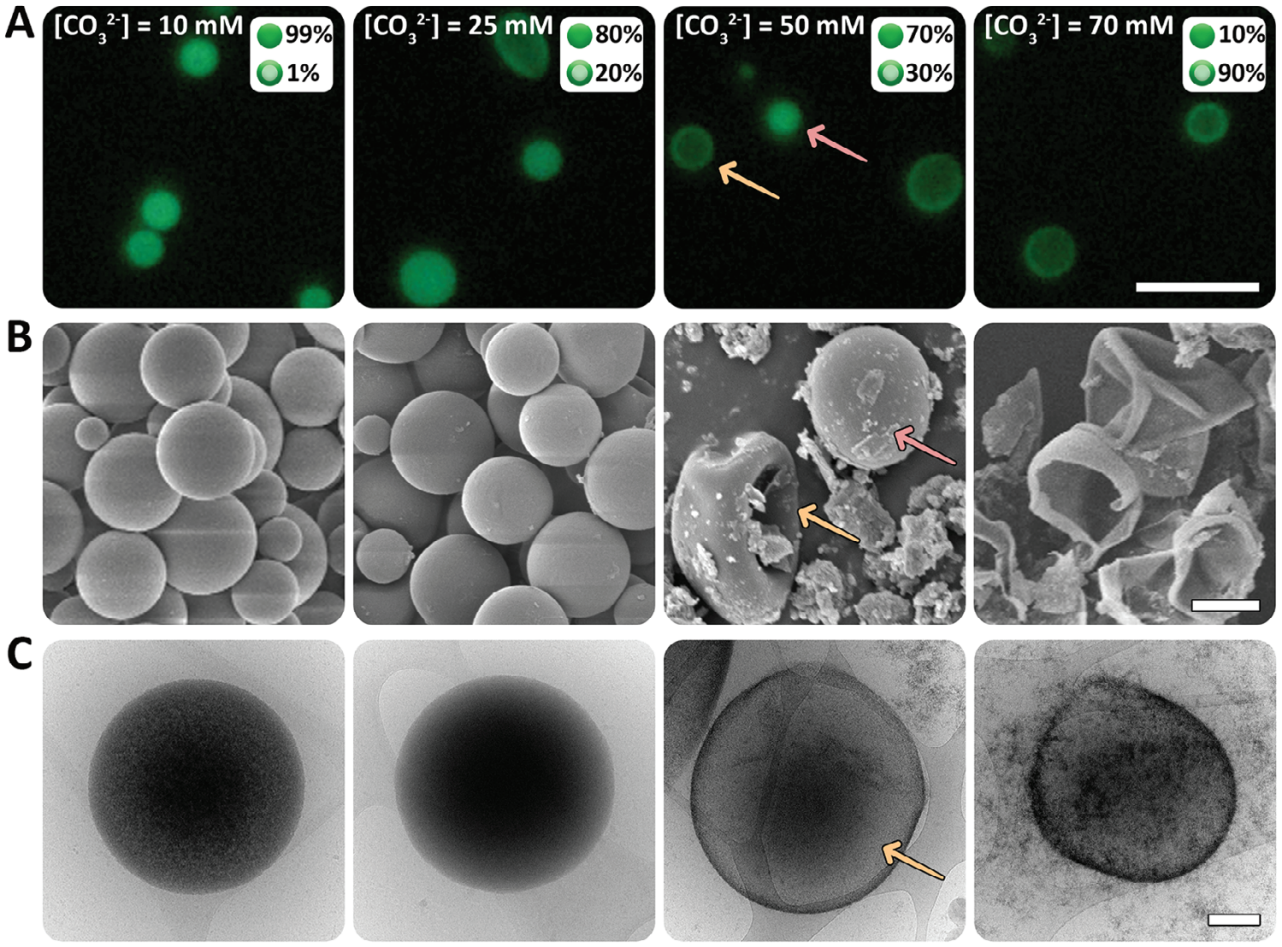
ACC formation through the diffusion of CO_3_^2−^ into PAA‐Ca dense droplets. A) Fluorescence images of PAA‐Ca‐CO_3_ spheres. The core–shell spheres (orange arrows) are dominant over the filled spheres (pink arrows) at higher [CO_3_^2−^]. The scale bar for all images is 20 µm. B) SEM images of dried samples. At 50 mm CO_3_^2^, we see both normal spheres and collapsed core–shell spheres, while at 70 mm we see mostly collapsed spheres. The scale bar for all images is 1 µm. C) Cryo‐TEM images of the ACC spheres in their native state show a similar trend. The scale bar for all images is 200 nm.

To investigate the kinetics of carbonate transport into the PAA‐Ca droplets, 50 mm of CO_3_^2−^ was added sequentially in increments of 10 mm, and the composition of the spheres was analyzed with EDS after each dose of carbonate addition. An increasing sodium content with carbonate addition points to the role of salt in maintaining charge balance (Figure S13, Suppporting Information), as well as the potential protonation of the polymer. The elemental composition of the samples where the carbonate was added sequentially was similar to those where carbonate was added instantaneously (Figure S14, Suppporting Information). This suggests a diffusion‐controlled process where the diffusion of CO_3_^2−^ into the PAA‐Ca dense droplets follows equilibrium dynamics and is not sensitive to specific mixing rates. To directly observe the effect of carbonate diffusion into the PAA‐Ca droplets, we analyzed the fluorescence of labeled PAA in the PAA‐Ca‐CO_3_ droplets. At 10 mm CO_3_^2−^, ≈99% of the ACC spheres looked isotropic (**Figure** **4**A). When the concentration was raised to 25 mm, ≈20% of the spheres had a core–shell structure. At 50 mm, it was ≈70%, and at even higher [CO_3_^2−^], 70 mm, the percentage of the core–shell spheres rose to ≈90%. The core–shell morphologies manifested in deformed spheres in SEM imaging of dried samples, and cryo‐TEM showed the core–shell architecture in its native hydrated conditions (Figure 4B,C). The correlation between higher [CO_3_^2−^] and the core–shell morphology indicates that high diffusion gradients lead to fast solidification of the hybrid‐ACC phase close to the phase boundary, which limits transport to the inner part of the droplet.

**Figure 5:**
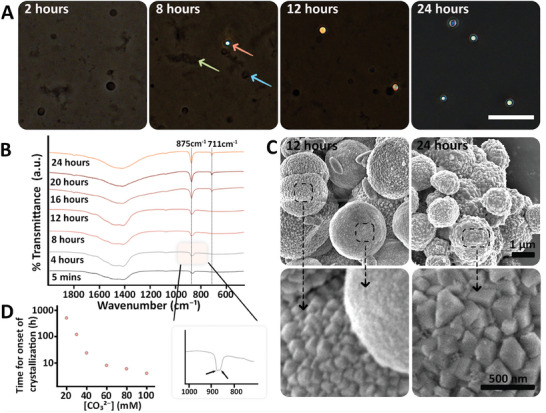
Crystallization of PAA‐Ca‐CO_3_ dense droplets over time. A) Light microscope images of samples with 60 mm CO_3_^2‐^ show the time‐dependent evolution of birefringence spheres from ACC spheres. The orange arrow points to a birefringent sphere, the blue arrow to an amorphous sphere, and the green one to aggregated nanoparticles. The scale bar for all images is 20 µm. B) Time series of FT‐IR spectra from the 60 mm CO_3_^2‐^ samples showing the continuous crystallization process. The first appearance of an 875 cm^−1^ peak was noted as the time for the onset of crystallization (Inset). C) SEM images of these samples after 12 and 24 h. High‐magnification images show the characteristic surfaces of spheres before and after crystallization. D) Initiation of crystallization in PAA‐Ca‐CO_3_ droplets synthesized with different [CO_3_^2−^].

Lastly, we investigated the stability of the amorphous PAA‐Ca‐CO_3_ spheres by following the time period after carbonate addition until the emergence of birefringence in optical microscopy, indicating their transformation to a crystalline phase. Spheres formed with 60 mm CO_3_^2−^ showed visible birefringence after 8 h. The fraction of crystalline spheres kept increasing until all the spheres had gradually crystallized after 24 h (**Figures** **5**A and S15, Suppporting Information). Crystallization was evident in the faceted morphologies imaged with SEM (Figure 5C). The crystallization process was also studied with FT‐IR, where the onset of the crystalline calcite peak at 875 cm^−1^ was first observed after 4 h, followed by a complete transformation to the crystalline spectrum (Figure 5B). We used the appearance of the 875 cm^−1^ peak as an indication for the onset of crystallization. Analyzing time‐dependent spectra of other samples that formed under different [CO_3_^2−^] showed that higher [CO_3_^2−^] leads to faster crystallization (Figure 5D). It is important to note that only in the sample with 60 mm CO_3_^2−^ the crystals had sizes reminiscent of the amorphous spheres. In other conditions, the products were crystals of variable sizes and shapes, indicating accompanying processes in the system, such as dissolution and recrystallization (Figure S16, Suppporting Information).

The picture that emerges from the observations of this condensate‐mediated ACC precipitation is a manifestation of several concepts, such as multi‐step crystallization, LLPS, and metastable precursors. All of which are widely discussed in the fields of biomineralization and bio‐inspired materials.^[^ ^5^, ^6^, ^17^, ^27^, ^40^ ^]^ An important difference from other ACC precipitation reactions is that the additional intermediate phase, the calcium‐rich coacervate phase, is an equilibrium phase and not a kinetic intermediate. This facilitates the gradual elevation of carbonate concentration in the condensate via diffusion, leading to the non‐stoichiometric Ca^2+^ to carbonate ratios in the condensates.

The nanoparticles that form in the dilute phase are reminiscent of ACC precipitating in the presence of low additive concentrations.^[^ ^41^ ^]^ They have the characteristic granular morphology and the stoichiometric CaCO_3_ composition. On the other hand, the dense droplets display a wider set of properties, which are characteristic of PILP systems.^[^ ^20^, ^28^ ^]^ First, they start as liquid and transform into a solid, which explains why various studies reached disparate views on the rheology of such materials.^[^ ^26^, ^28^, ^42^ ^]^ Second, their composition is tunable and non‐stoichiometric, as required by a diffusion‐driven chemical reaction. It will be very interesting to investigate how different polymers affect the final product. PAA is a generic polymer whose only important property for this system is the negative charge. Other negatively charged polymers, such as biogenic glycoproteins, were shown to relate to specific features of the process and its products.^[^ ^43^, ^44^, ^45^ ^]^

The non‐stoichiometric composition is an important trait of this system as it allows to regulate the stability of the ACC. The dilute phase ACC forms via nucleation and growth, yielding a stoichiometric disordered phase. However, the ACC in the condensate forms via diffusion, probably when carbonate ions replace the carboxylic acid residues of the PAA in coordinating Ca^2+^ ions. This allows for a gradual compositional change, similar to a solid‐solution,^[^ ^46^ ^]^ which in turn causes a gradual change in stability, from a more stable ACC rich in PAA to a less stable and inorganic‐rich ACC. This serves as an internal regulator to temporally tune the stability of the system and the onset of crystallization. In this system, the presence of the macromolecules not only increases the kinetic barrier for calcite nucleation, similar to their effect in dilute conditions, but it serves a second role where the kinetics of carbonate diffusion across the LLPS interface causes a gradual rise in supersaturation levels within the condensates.

The condensate‐mediated ACC formation is relevant to recent discussions over the role of LLPS in calcium carbonate precipitation. Many studies referred to the possibility that LLPS between the calcium and carbonate species precedes the first solid CaCO_3_ phase, from pre‐nucleation clusters to larger colloids.^[^ ^17^, ^18^, ^25^, ^27^ ^]^ These ideas were discussed regarding systems containing polymers, but usually, the dense phase consisted of both calcium and carbonate species. This report separates the LLPS of the polymer and calcium from the subsequent introduction of carbonate, pointing to a strategy how to simplify the basic understanding of such complex systems.

The coacervate‐based system for ACC precipitation might be highly relevant to biological systems, where much has been investigated regarding the transformation of the amorphous precursor to a crystalline phase. Still, the preceding step of how the amorphous precursor itself forms from soluble components is perplexing. This is because ion transport rates through physiological processes are relatively low and accommodate only slow increases in supersaturation levels.^[^ ^47^ ^]^ Such slow system dynamics can lead to the formation of the stable crystalline phase in supersaturation levels where calcite is supersaturated and ACC is not. In laboratory syntheses, this challenge is usually overcome by fast quenching of reactions between very concentrated solutions, even though other strategies were demonstrated.^[^ ^48^, ^49^ ^]^ This allows to bring the system to kinetic traps, where the amorphous phase forms in far‐from‐equilibrium conditions.^[^ ^5^ ^]^ These characteristics are incompatible with physiological conditions, and we propose that the principles of the presented process can underlie the chemical process that organisms use to precipitate metastable ACC.

This notion that cellular condensates are precursors in mineral formation is in line with the recent breakthroughs in biocondensates research.^[^ ^50^, ^51^ ^]^ Biocondensates are being recognized as pivotal drivers of many biochemical reactions in cells, making it very attractive that evolution took advantage of the same principles in the formation of inorganic solids. The variability in biogenic negatively‐charged macromolecules associated with biominerals that form via an amorphous precursor may indicate that evolutionary adaptations for the properties of the process and its resulting materials can be used to tailor structure‐function properties.

## 3 Conclusion

Phase‐separated polymer‐Ca solutions provide a flexible environment to control the formation of ACC. In the dilute phase, ACC precipitates via a supersaturation‐driven secondary phase separation. However, the condensate droplets uptake carbonate via diffusion dynamics that proceed gradually toward chemical equilibrium with the dilute phase. The fact that the resulting supersaturation in the condensate can lead to calcium carbonate crystallization is reminiscent of the physiological process that organisms use to form functional organic–inorganic materials.

## Conflict of Interest

The authors declare no conflict of interest.

## Author Contributions

D.P designed and performed experiments and analyzed the data. N.V contributed to data acquisition, processing, and analysis. P.B and L.A. collected cryo‐TEM data. I.K.A supervised EDS data collection and analysis. A.G supervised research and wrote the manuscript with input from all authors.

## Supporting information



Supporting Information

## Data Availability

The data that support the findings of this study are available in the supplementary material of this article.

## References

[1] J. S. Du , Y. Bae , D. Yoreo , Nat. Rev. Mater. 2024, 9, 229.

[2] D. Gebauer , S. E. Wolf , J. Am. Chem. Soc. 2019, 141, 4490. 30753066 10.1021/jacs.8b13231

[3] J. J. De Yoreo , P. G. Vekilov , Rev. Mineral. Geochem. 2003, 54, 57.

[4] F. C. Meldrum , H. Cölfen , Chem. Rev. 2008, 108, 4332. 19006397 10.1021/cr8002856

[5] J. J. De Yoreo , P. U. P. A. Gilbert , N. A. J. M. Sommerdijk , R. L. Penn , S. Whitelam , D. Joester , H. Zhang , J. D. Rimer , A. Navrotsky , J. F. Banfield , A. F. Wallace , F. M. Michel , F. C. Meldrum , H. Cölfen , P. M. Dove , Science 2015, 349, aaa6760. 26228157 10.1126/science.aaa6760

[6] P. G. Vekilov , Nanoscale 2010, 2, 2346. 20936214 10.1039/c0nr00628a

[7] S. Weiner , L. Addadi , Annu. Rev. Mater. Res. 2011, 41, 21.

[8] P. U. P. A. Gilbert , K. D. Bergmann , N. Boekelheide , S. Tambutté , T. Mass , F. Marin , J. F. Adkins , J. Erez , B. Gilbert , V. Knutson , M. Cantine , J. O. Hernández , A. H. Knoll , Sci. Adv. 2022, 8, eabl9653. 35263127 10.1126/sciadv.abl9653 PMC8906573

[9] L. Addadi , S. Raz , S. Weiner , Adv. Mater. 2003, 15, 959.

[10] J. H. E. Cartwright , A. G. Checa , J. D. Gale , D. Gebauer , Angew. Chem. Inter. Ed. 2012, 51, 11960. 10.1002/anie.201203125 23124964

[11] P. J. M. Smeets , A. R. Finney , W. J. E. M. Habraken , F. Nudelman , H. Friedrich , J. Laven , J. J. De Yoreo , P. M. Rodger , N. A. J. M. Sommerdijk , Proc. Natl. Acad. Sci. USA 2017, 114, E7882. 28874584 10.1073/pnas.1700342114 PMC5617248

[12] D. Gebauer , A. Völkel , H. Cölfen , Science 2008, 322, 1819. 19095936 10.1126/science.1164271

[13] D. Gebauer , J. D. Gale , H. Cölfen , Small. 2022, 18, 2107735. 10.1002/smll.202107735 35678091

[14] B. Jin , Y. Chen , H. Pyles , M. D. Baer , B. A. Legg , Z. Wang , N. M. Washton , K. T. Mueller , D. Baker , G. K. Schenter , C. J. Mundy , D. Yoreo , Nat. Mater. 2024, 24, 125. 39448841 10.1038/s41563-024-02025-5

[15] A. F. Wallace , L. O. Hedges , A. Fernandez‐Martinez , P. Raiteri , J. D. Gale , G. A. Waychunas , S. Whitelam , J. F. Banfield , D. Yoreo , Science 2013, 341, 885. 23970697 10.1126/science.1230915

[16] S. E. Wolf , J. Leiterer , M. Kappl , F. Emmerling , W. Tremel , J. Am. Chem. Soc. 2008, 130, 12342. 18717561 10.1021/ja800984y

[17] J. T. Avaro , S. L. P. Wolf , K. Hauser , D. Gebauer , Angew. Chem. Inter. Ed. 2020, 59, 6155. 10.1002/anie.201915350 PMC7187218 31943581

[18] M. Faatz , F. Gröhn , G. Wegner , Adv. Mater. 2004, 16, 996.

[19] Z. Liu , C. Shao , B. Jin , Z. Zhang , Y. Zhao , X. Xu , R. Tang , Nature 2019, 574, 394. 31619792 10.1038/s41586-019-1645-x

[20] L. B. Gower , Chem. Rev. 2008, 108, 4551. 19006398 10.1021/cr800443h PMC3652400

[21] J. Aizenberg , J. Hanson , T. F. Koetzle , S. Weiner , L. Addadi , J. Am. Chem. Soc. 1997, 119, 881.

[22] G. Falini , S. Albeck , S. Weiner , L. Addadi , Science 1996, 271, 67.

[23] Y.‐W. Wang , Y.‐Y. Kim , C. J. Stephens , F. C. Meldrum , H. K. Christenson , Cryst. Growth Des. 2012, 12, 1212.

[24] Y. Politi , J. Mahamid , H. Goldberg , S. Weiner , L. Addadi , CrystEngComm 2007, 9, 1171.

[25] P. I. Schodder , M. B. Gindele , A. Ott , M. Rückel , R. Ettl , V. Boyko , M. Kellermeier , Phys. Chem. Chem. Phys. 2022, 24, 9978. 35319032 10.1039/d1cp05606a

[26] Y. Xu , K. C. H. Tijssen , P. H. H. Bomans , A. Akiva , H. Friedrich , A. P. M. Kentgens , N. A. J. M. Sommerdijk , Nat. Commun. 2018, 9, 2582. 29968713 10.1038/s41467-018-05006-w PMC6030133

[27] F. Sebastiani , S. L. P. Wolf , B. Born , T. Q. Luong , H. Cölfen , D. Gebauer , M. Havenith , Angew. Chem. Inter. Ed. 2017, 56, 490. 10.1002/anie.201610554 28029201

[28] L. B. Gower , D. J. Odom , J. Cryst. Growth 2000, 210, 719.

[29] Y. Oaki , S. Kajiyama , T. Nishimura , H. Imai , T. Kato , Adv. Mater. 2008, 20, 3633.

[30] M. B. Gindele , S. Nolte , K. M. Stock , K. Kebel , D. Gebauer , Adv. Mater. 2023, 35, 2300702. 10.1002/adma.202300702 36971032

[31] A. T. Rowland , D. N. Cacace , N. Pulati , M. L. Gulley , C. D. Keating , Chem. Mater. 2019, 31, 10243.

[32] S. Sun , L. B. Mao , Z. Lei , S. H. Yu , H. Cölfen , Angew. Chem. Inter. Ed. 2016, 55, 11765. 10.1002/anie.201602849 27444970

[33] P. Kaempfe , V. R. Lauth , T. Halfer , L. Treccani , M. Maas , K. Rezwan , J. Am. Ceram. Soc. 2013, 96, 736.

[34] T. Mittag , R. V. Pappu , Mol. Cell 2022, 82, 2201. 35675815 10.1016/j.molcel.2022.05.018 PMC9233049

[35] C. Fernández‐Rico , T. Sai , A. Sicher , R. W. Style , E. R. Dufresne , JACS Au 2022, 2, 66. 35098222 10.1021/jacsau.1c00443 PMC8790737

[36] S. F. Banani , H. O. Lee , A. A. Hyman , M. K. Rosen , Nat. Rev. Mol. Cell Biol. 2017, 18, 285. 28225081 10.1038/nrm.2017.7 PMC7434221

[37] Y. Shin , C. P. Brangwynne , Science 2017, 357. 10.1126/science.aaf4382 28935776

[38] A. A. Hyman , C. A. Weber , F. Jülicher , Ann. Rev. Cell Dev. Biol. 2014, 30, 39. 25288112 10.1146/annurev-cellbio-100913-013325

[39] B. Gouveia , Y. Kim , J. W. Shaevitz , S. Petry , H. A. Stone , C. P. Brangwynne , Nature 2022, 609, 255. 36071192 10.1038/s41586-022-05138-6

[40] I. Polishchuk , A. A. Bracha , L. Bloch , D. Levy , S. Kozachkevich , Y. Etinger‐Geller , Y. Kauffmann , M. Burghammer , C. Giacobbe , J. Villanova , G. Hendler , C. Y. Sun , A. J. Giuffre , M. A. Marcus , L. Kundanati , P. Zaslansky , N. M. Pugno , P. U. P. A. Gilbert , A. Katsman , B. Pokroy , Science 2017, 358, 1294. 29217569 10.1126/science.aaj2156

[41] Z. Zou , W. J. E. M. Habraken , L. Bertinetti , Y. Politi , A. Gal , S. Weiner , L. Addadi , P. Fratzl , Adv. Mater. Interfaces 2017, 4, 1600076.

[42] C. Shao , H. Pan , J. Tao , K. R. Cho , R. Tang , L. B. Gower , J. J. De Yoreo , Chem. Commun. 2024, 60, 3950. 10.1039/d4cc00449c 38498350

[43] G. Jain , M. Pendola , A. Rao , H. Cölfen , J. S. Evans , Biochemistry 2016, 55, 4410. 27426695 10.1021/acs.biochem.6b00619

[44] J. L. Arias , M. S. Fernández , Chem. Rev. 2008, 108, 4475. 18652513 10.1021/cr078269p

[45] S. Albeck , S. Weiner , L. Addadi , Chem.‐Eur. J. 1996, 2, 278.

[46] N. Pinsk , A. Wagner , L. Cohen , C. J. H. Smalley , C. E. Hughes , G. Zhang , M. J. Pavan , N. Casati , A. Jantschke , G. Goobes , K. D. M. Harris , B. A. Palmer , J. Am. Chem. Soc. 2022, 144, 5180. 35255213 10.1021/jacs.2c00724 PMC8949762

[47] K. Kahil , S. Weiner , L. Addadi , A. Gal , J. Am. Chem. Soc. 2021, 143, 21100. 34881565 10.1021/jacs.1c09174 PMC8704196

[48] C. C. Tester , R. E. Brock , C.‐H. Wu , M. R. Krejci , S. Weigand , D. Joester , CrystEngComm 2011, 13, 3975.

[49] F. C. Meldrum , O'C. Shaughnessy , Adv. Mater. 2020, 32, 2001068. 10.1002/adma.202001068 32583495

[50] A. Gal , R. Wirth , J. Kopka , P. Fratzl , D. Faivre , A. Scheffel , Science 2016, 353, 590. 27493186 10.1126/science.aaf7889

[51] L. Krounbi , K. Hedderick , Z. Eyal , L. Aram , E. Shimoni , L. A. Estroff , A. Gal , Chem. Mater. 2021, 33, 3534.

