# Supplementary material for: Non‐Stoichiometric Amorphous Calcium Carbonate Forms in Macromolecular Condensates via Interphase Diffusion
Source: Small. 2025 Jan 16;21(10):2411965. doi: 10.1002/smll.202411965 (PMC11899510; doi:10.1002/smll.202411965)
Supplement: Supplementary file 1 — Supporting Information [file SMLL-21-2411965-s001.docx]

**Supporting Information**

**Non-stoichiometric amorphous calcium carbonate forms in macromolecular condensates via interphase diffusion**

*Debojit Paul*^1^*, Neta Varsano*^2^*, Protap Biswas*^1^*, Ifat Kaplan-Ashiri*^2^*, Lior Aram*^1^*, and Assaf Gal*^1^***

**Materials and methods.**

**Reagents.** Calcium chloride dihydrate (ACS reagent, ≥99%), sodium carbonate anhydrous (EMSURE), Poly (acrylic acid, sodium salt) (Mw ~1200 kDa, 45 wt. % in H_2_O), and Poly (acrylic acid) (Mw ~100 MDa, 35 wt. % in H_2_O) were all purchased from Sigma-Aldrich and used without any further purification. The concentrations of the polymers were calculated in terms of the monomer units. Milli-Q water (resistivity: 18.2 MΩ·cm at 25 ℃) was used for solution preparation. For pH adjustments, 1 M HCl and 1 M NaOH solutions were used. For fluorescent labelling, BODIPY FL amine (Mw 426.74 Da) was purchased from Lumiprobe LTD Israel (2183473-03-0), N-(3-Dimethylaminopropyl)-N′-ethylcarbodiimide hydrochloride (EDAC) and dialysis tubing cellulose membrane (Mw cut-off = 14,000) were purchased from Sigma-Aldrich.

**Synthesis of PAA-Ca-CO_3_.** The process begins with preparing the PAA (Mw ~1200 kDa) and CaCl_2_.2H_2_O stock solutions at concentrations of 640 mM (10 times dilution of the purchased solution) and 1 M respectively in Milli-Q water, pH adjusted to 9. After mixing the PAA and Ca^2+^ solutions, the condensates are given time to fuse and grow, after which, CO_3_^2-^ is introduced from the stock solution, 1 M Na_2_CO_3_ in Milli-Q water. The pH of the resulting solution remains close to 9. The resulting precipitate is either analyzed in the native form or in the powdered form upon washing and lyophilization. A typical washing procedure of the samples involved centrifugation at 15000 g for 2 minutes, followed by decantation of the liquid part. The pellets were resuspended in fresh Milli-Q water after every round. The process was repeated two times to wash away any unreacted phases. The washed pellets were lyophilized to obtain the samples as dried powders.

**Fluorescent tagging of BODIPY FL Amine to Polyacrylic acid by EDAC coupling.** For the preparation of stock solutions, 1 mg of BODIPY FL Amine was dissolved in 200 μL of MilliQ H_2_O (concentration of 0.0117 M), PAA (Mw ~100 MDa) was diluted using MilliQ H_2_O to give a stock solution of 556 mM. 10 mg EDAC was dissolved in 1 ml MilliQ H_2_O. Phosphate buffer, 0.1 M, 100 ml of pH=7.8 was prepared using K_2_HPO_4_ and KH_2_PO_4_. In the coupling reaction, 180 μL of PAA from the stock solution was taken in an Eppendorf microtube (1.5 mL), to yield a concentration of 100 mM (in 1 mL). The solution was made basic (pH=8) by adding 5 μL of NaOH (1 M). Subsequently, 320 μL of the phosphate buffer was added, followed by 470 μL of EDAC stock solution. The resulting solution was vortex shaken for 5 mins. Simultaneously, 20 μL of dye from its stock solution was taken, and 5 μL of NaOH (1 M) was added to make it basic. It was then added to the PAA+EDAC solution and the resulting solution was vortexed for 10 minutes. The reaction mixture was left overnight to rotate at a slow speed in the rotator. Finally, the solution was dialyzed for 24 hours, and the water was replaced 3 times.

**Fluorescence and Light microscope imaging.** 15μL of solution sample was pipetted on a glass slide and placed a cover slip over it. The samples were observed in a Nikon Upright Optical Microscope, Eclipse Ni-U. The fluorescence images were obtained using excitation at 488 nm and detection at 510 nm.

**Dynamic light scattering (DLS).** A Zetasizer (Nano ZSP, Malvern Instruments, United Kingdom) equipped with a 633 nm laser was used to measure particle sizes in real time. The particle sizes were determined by intensity distribution and presented as the average values of three replicate measurements.

**Scanning Electron Microscope (SEM).** The lyophilized samples were then mounted on SEM stubs with the help of a carbon tape, which were then coated with 5 nm Iridium (Compact Coating Unit, CCU-010, Safematic), and imaged using SEM (Sigma 500, Zeiss).

**Energy Dispersive X-Ray Spectroscopy (EDS).** Measurements were performed in Zeiss Ultra 55 using the Bruker FlatQUAD - four quadrants retractable detector. The accelerating voltage was set to 6 KV and the EDS was set to windowless mode to obtain optimum signals in the form of counts per second from the samples for efficient measurements. The samples were prepared and coated the same way as for SEM imaging.

**Fourier Transform Infrared (FT-IR) spectroscopy.** The lyophilized samples were measured by preparing KBr pellets on a Thermo Scientific Nicolet iS5 FT-IR Spectrometer. Data were analyzed with Origin 2018 version 95E, where deconvolution of the FT-IR peaks in the range of 1800-1180 cm^-1^ was performed. A total of six peaks were considered in this range to give the best possible fitting (Fig. S12). However, for the analysis, we considered the three important peaks, 1556 cm^-1^ (PAA); 1416 cm^-1^ and 1456 cm^-1^ (CO_3_^2-^) (Fig. 3A). In addition to the PAA-Ca-CO_3_ samples, pure PAA and ACC synthesized at low [PAA] were taken as controls.

**Thermogravimetric analysis (TGA).** Analyses of the lyophilized samples were performed on SDT Q600, TA Instruments, USA. Analyses were performed under an air atmosphere (injection rate of 100 mL/min) with a heating rate of 10 K/min. A typical measurement was 4 mg of lyophilized sample. For this, the sample preparation was scaled up to a higher volume (15 mL centrifuge tubes) and precipitates from several batches (~4-5) were collected.

**Cryo-Transmission Electron Microscopy (TEM).** 4 μL of sample solution was drop-casted on the carbon side of plasma-discharged Lacey/Carbon 200 mesh, Copper grids. The back-blotted grid was vitrified in liquid ethane using a Leica EM GP automatic plunger (Leica, Vienna, Austria) under 25 °C and 90% humidity conditions. The vitrified grids were transferred and kept immersed in liquid nitrogen until being used. Cryo-TEM imaging was performed on Talos Arctica (Thermo Fisher Scientific) with a Falcon 4i camera with an accelerating voltage of 200 kV.

**Supporting figures**


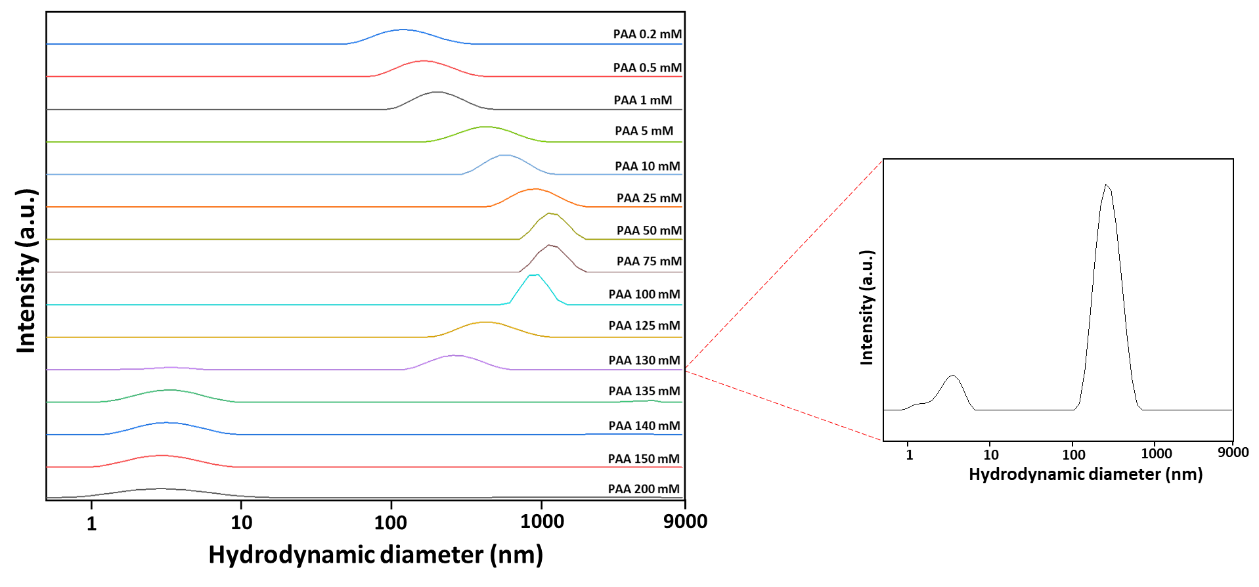
**Figure S1. Raw DLS datasets of PAA solutions mixed with 50 mM [Ca^2+^].** The peak maxima were used to plot the graph in Fig. 1 B. Magnification of the experiment at 130 mM PAA shows the presence of two peaks.

**
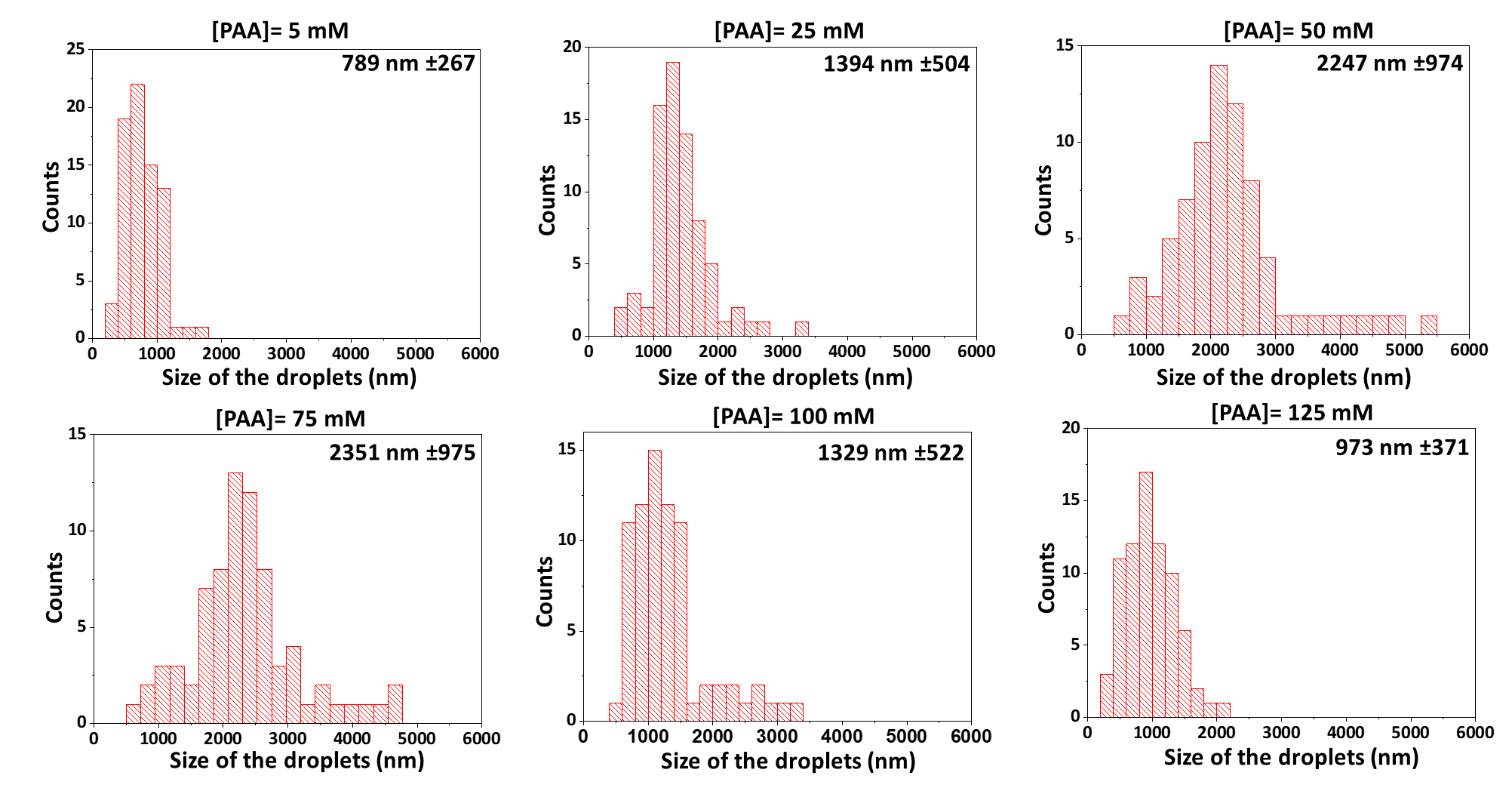
**

**Figure S2. Histograms of the phase-separated droplet size.** [Ca^2+^] was 50 mM, and [PAA] is indicated above each histogram. This quantifies the droplet size presented in Fig. 1C. 75 spheres were chosen in each condition to determine the mean sizes and their standard deviation (values incorporated as insets).


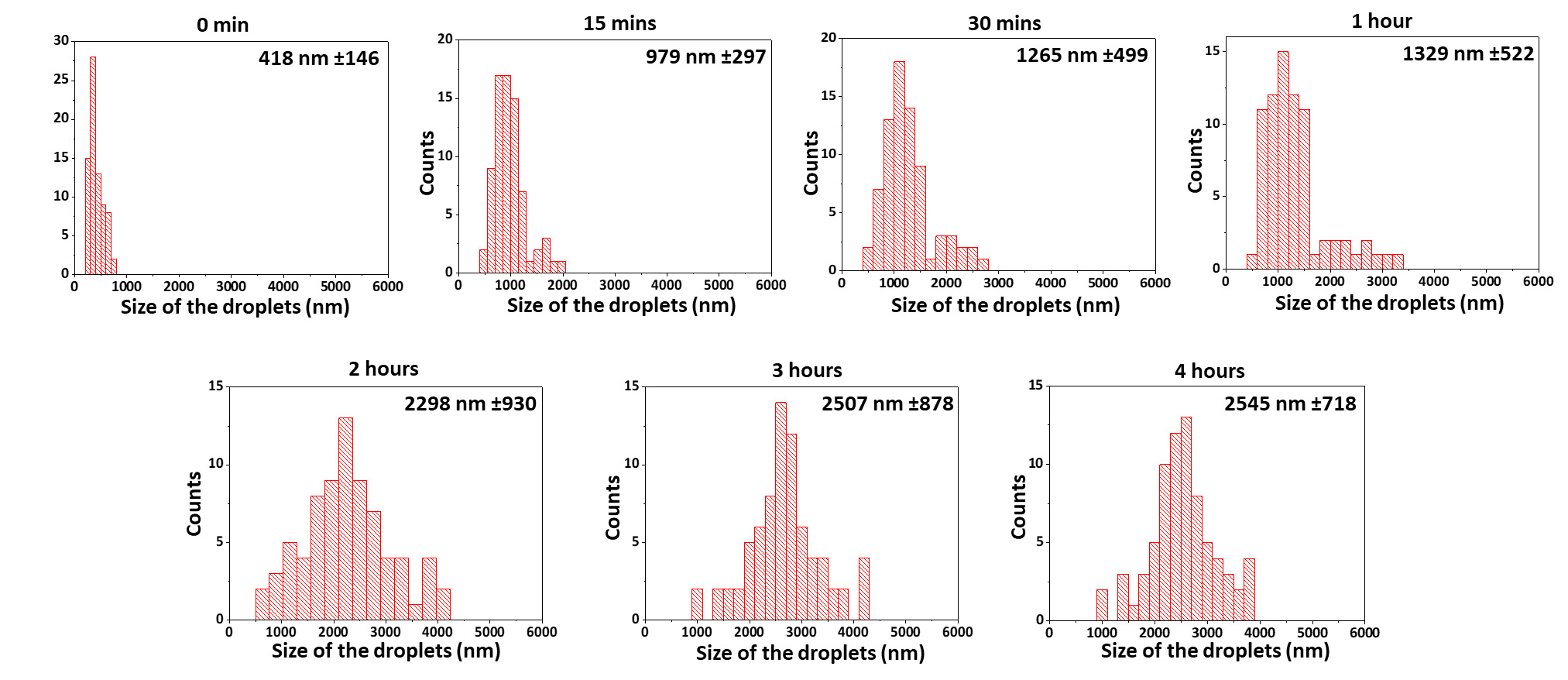


**Figure S3. Histograms representing the growth in size of the phase-separated droplets over time.** This quantifies the droplet size presented in Fig. 1D. 75 spheres were chosen in each time point to determine the mean sizes and their standard deviation (values incorporated as insets).

**
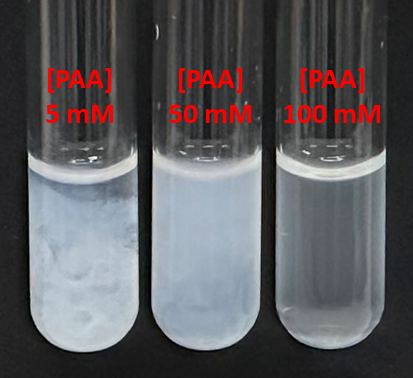
**

**Figure S4. Images of PAA-Ca mixtures after the addition of CO_3_^2-^.** Maximum turbidity was observed for low concentrations of PAA. At higher concentrations, there was no pronounced change in the already turbid solution.


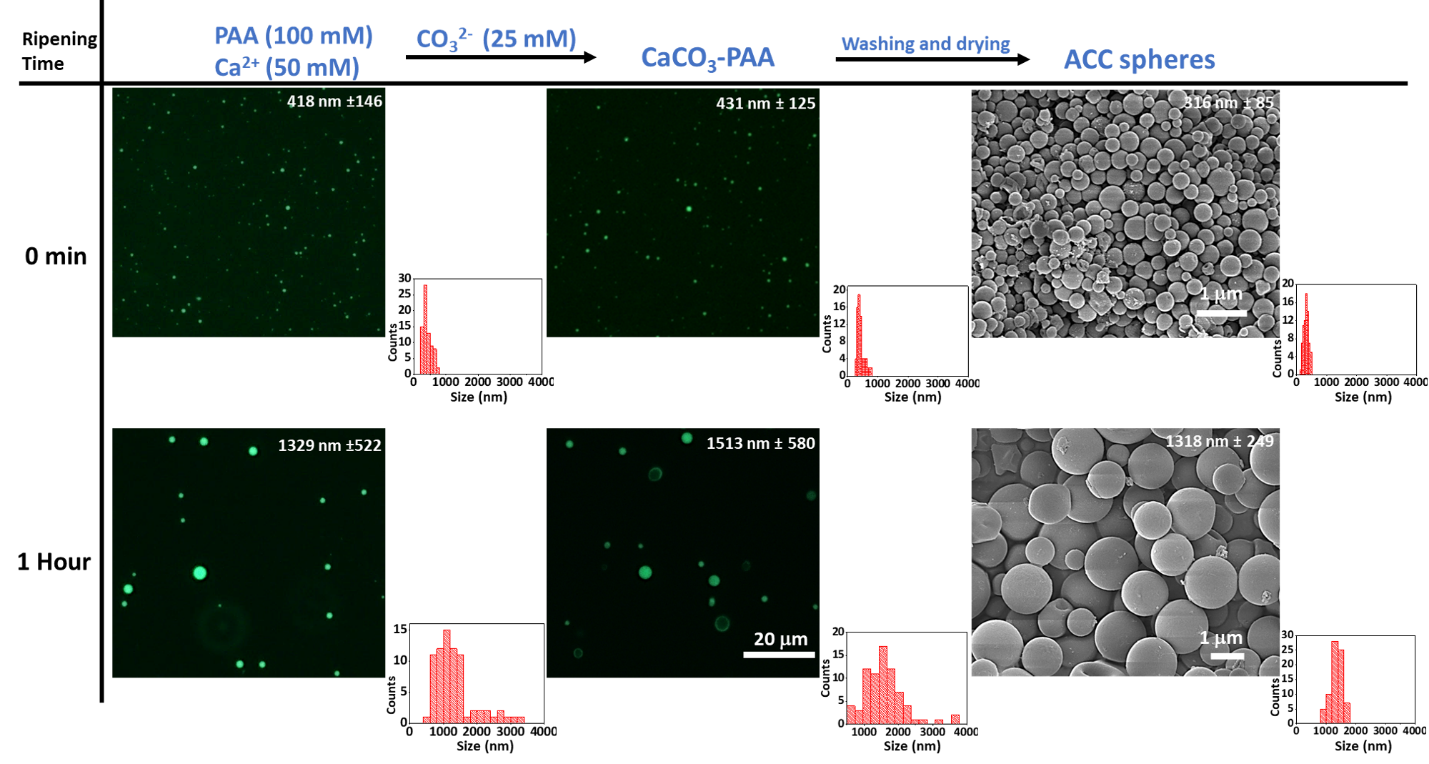
**Figure S5. Comparing the sizes and abundance of the PAA-Ca dense droplets and the PAA-Ca-CO_3_ spheres.** Two different time points in the coacervation process were chosen, yielding different sizes of the PAA-Ca droplets prior to the addition of CO_3_^2-^. The dense droplets and the ACC spheres have a comparable mean diameter (values incorporated as insets) both in the native and the dried states. All the fluorescence images have the same scale bar.


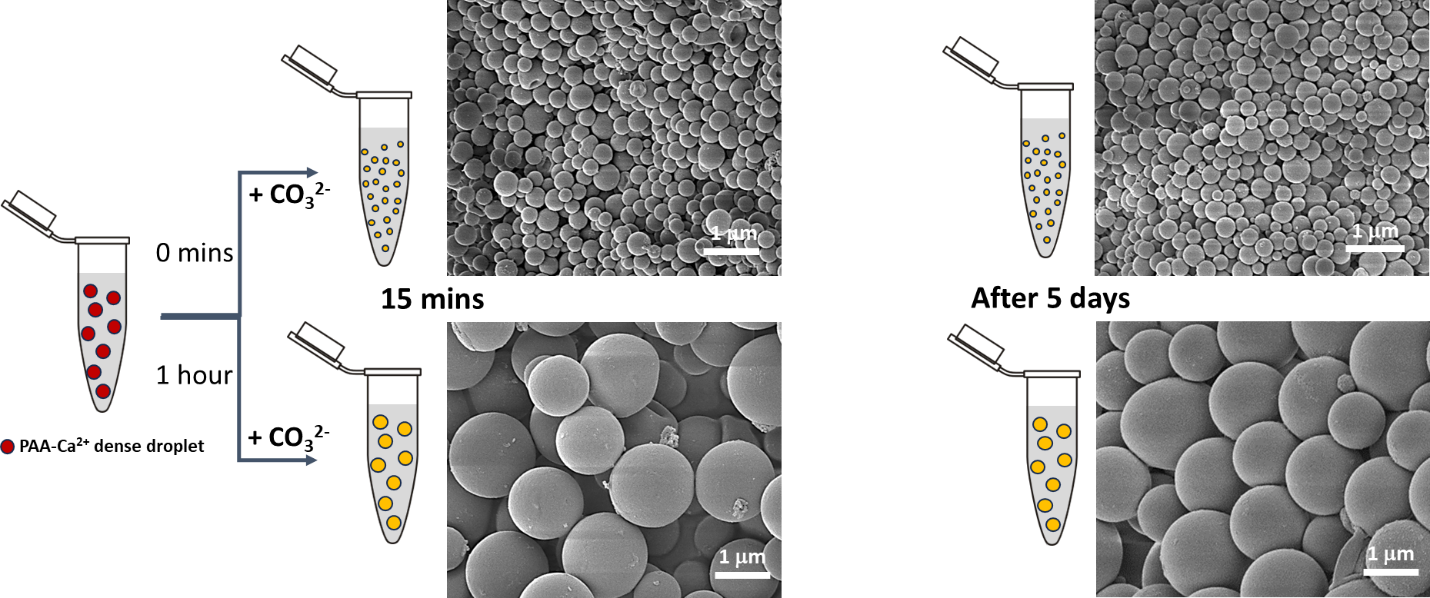


**Figure S6. Stability of the PAA-Ca-CO_3_ spheres in solution over time.** Two different sized PAA-Ca dense droplets obtained by the mixing of [PAA] 100 mM and [Ca^2+^] were taken, 0 minutes and 1 hour ripening time and [CO_3_^2-^] 25 mM was added to form ACC. They remained as ACC spheres without any change in size even after 5 days in solution.


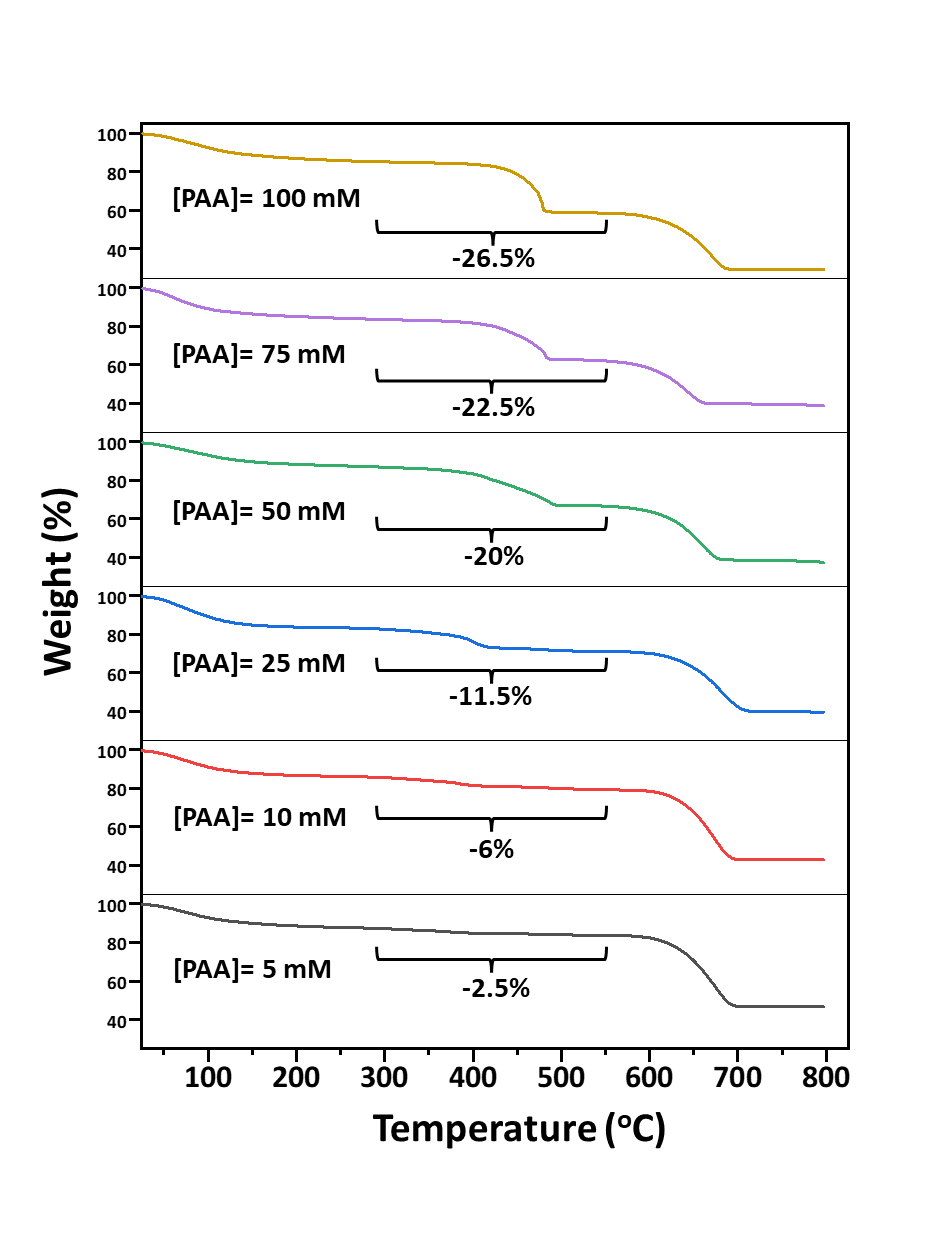


**Figure S7. TGA spectra of dried samples synthesized at different [PAA].** The weight loss due to the organic polymer PAA is in the temperature range of 300-550 ^o^C. The weight loss % has been labeled in each spectrum. A gradual increase in organic polymer content in the synthesized ACC samples is observed with increasing PAA concentration.


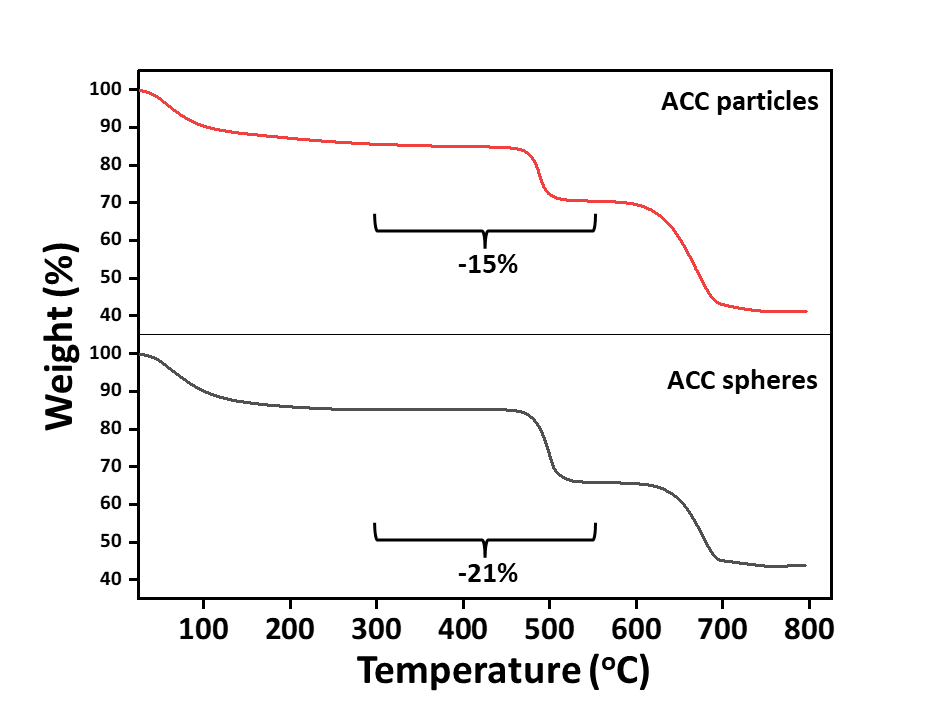


**Figure S8. TGA spectra of ACC particles and spheres separated gravimetrically.** It was possible to obtain relatively pure samples of nanoparticles and spheres from the initial mixture using slow centrifugation. The spheres evolving from the dense PAA-Ca droplets manifest a higher organic polymer content as compared to the nanoparticles formed from the dilute phase.


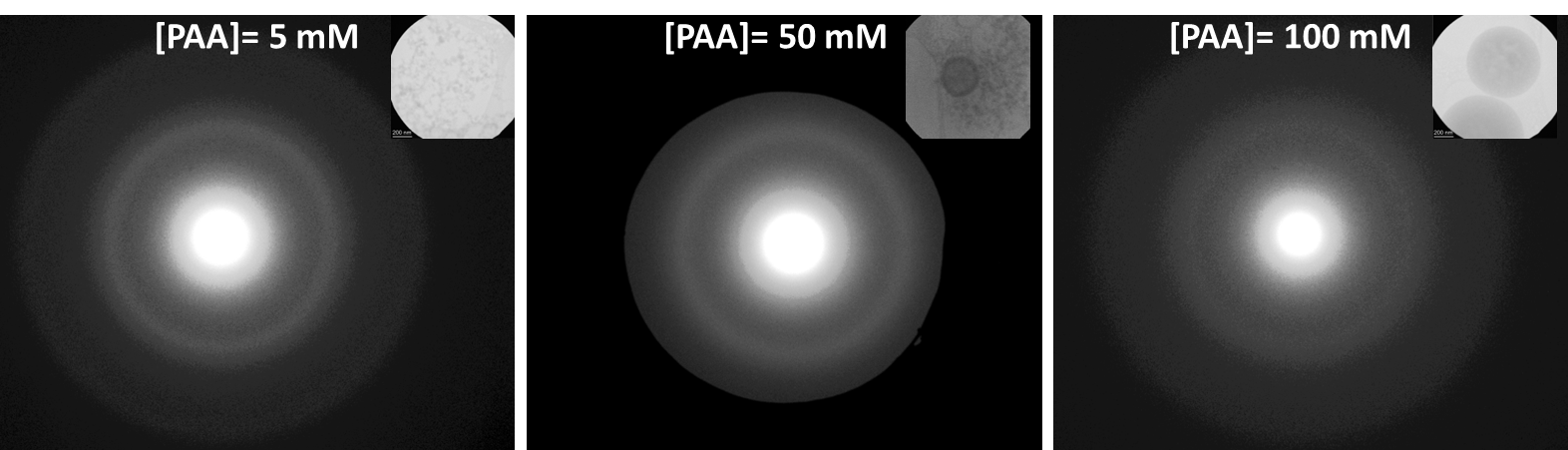


**Figure S9. Electron diffraction patterns of vitrified samples synthesized at different [PAA].** Insets show the selected area aperture diffraction image of the measured cryo sample. The diffused ring patterns demonstrate the amorphous nature of the samples.


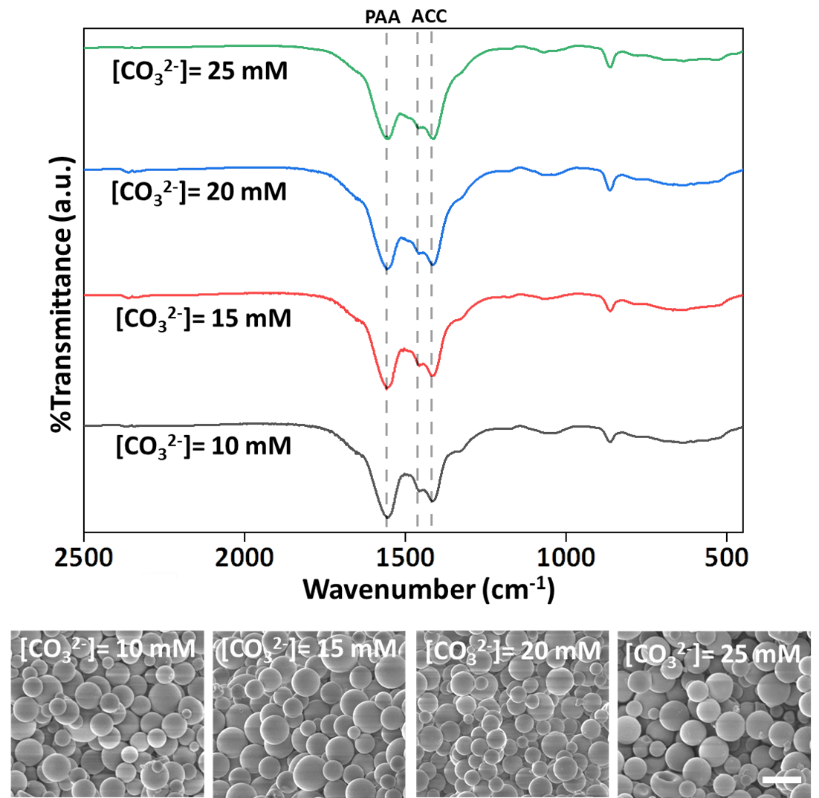


**Figure S10. ACC synthesized with 10-25 mM [CO_3_^2-^] at constant [PAA] 100 mM and [Ca^2+^] 50 mM.** Full FT-IR spectra of the samples presented in Fig. 3 and SEM images of the samples show that in all four different concentrations, the spheres are in abundance. The scale bar for all images is 2 μm.


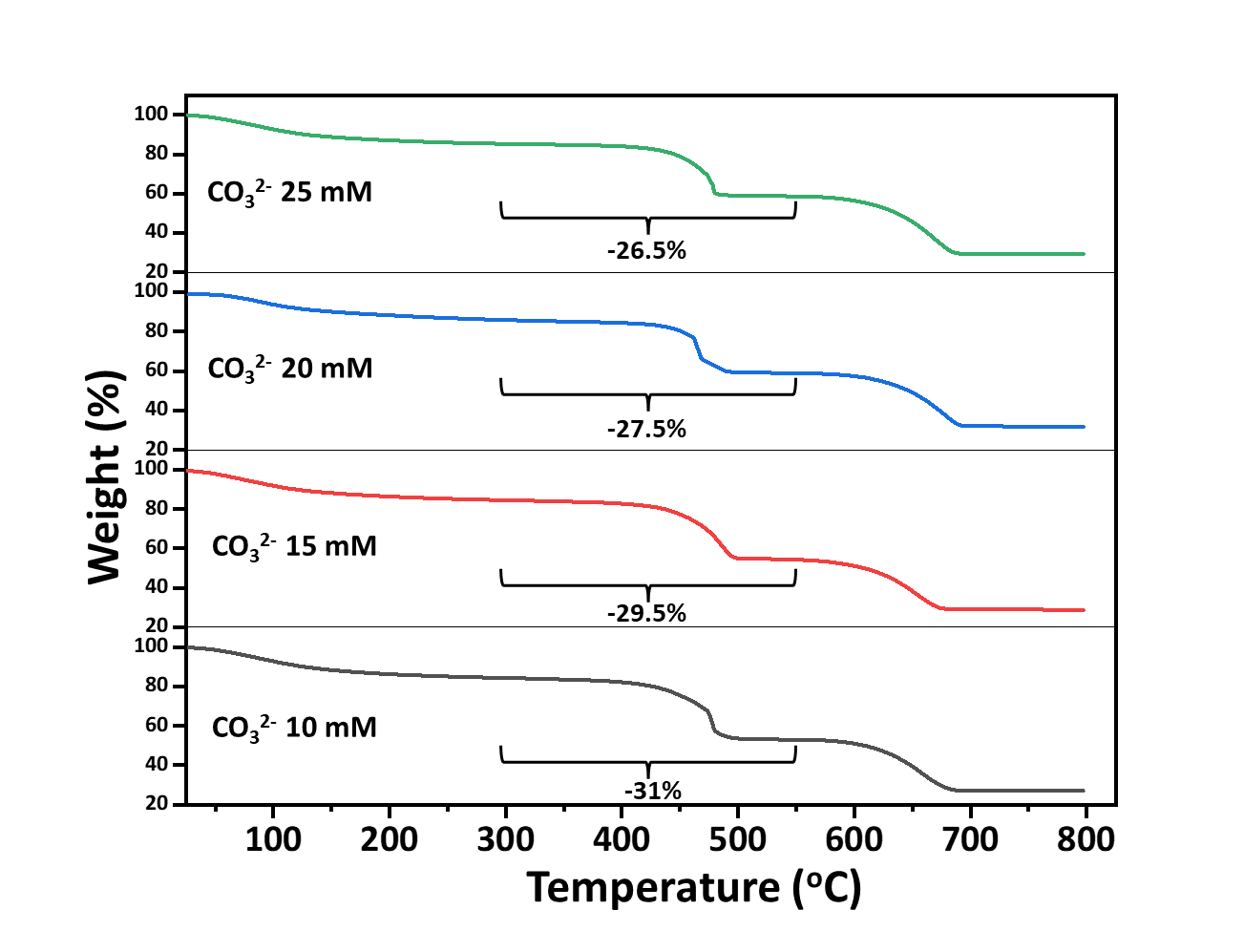


**Figure S11. TGA spectra of PAA-Ca-CO_3_ spheres synthesized at different [CO_3_^2-^].** The weight loss % due to the organic polymer PAA has been labeled in each spectrum. The observed trend suggests an increasing [CO_3_^2-^] that leads to a lowering of PAA content in the composition of the synthesized PAA-Ca-CO_3_ spheres.


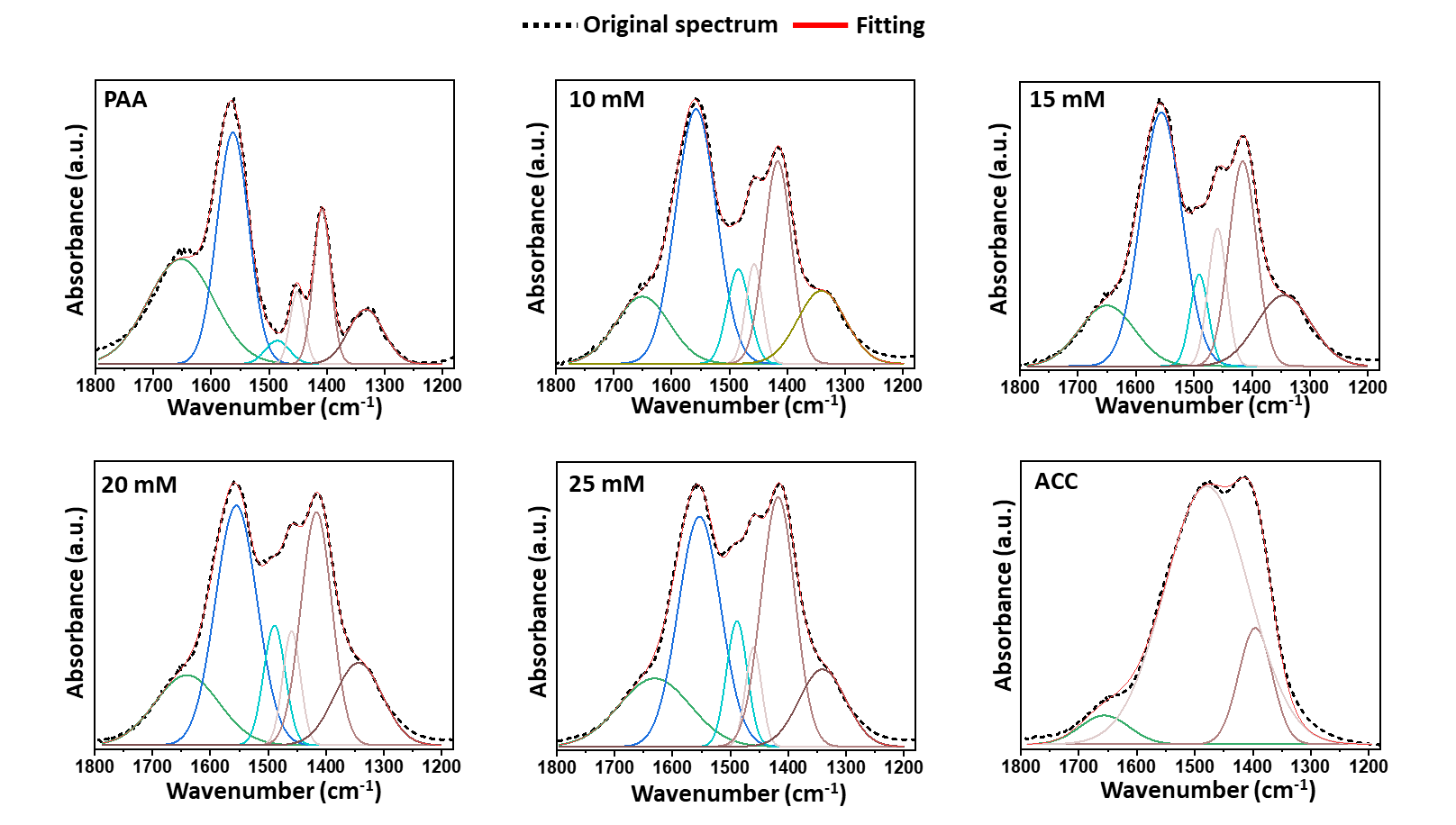


**Figure S12. Gaussian peak fits showing all the six peaks in the range 1800 cm^-1^ to 1180 cm^-1^.** In addition to the main two ACC peaks and one PAA peak, three additional Gaussians were used to reach a good fit. These are broader, lower intensity Gaussians (green, light blue, and brown). The locations of all 6 peaks were fixed and only their height and width were fitted.

**
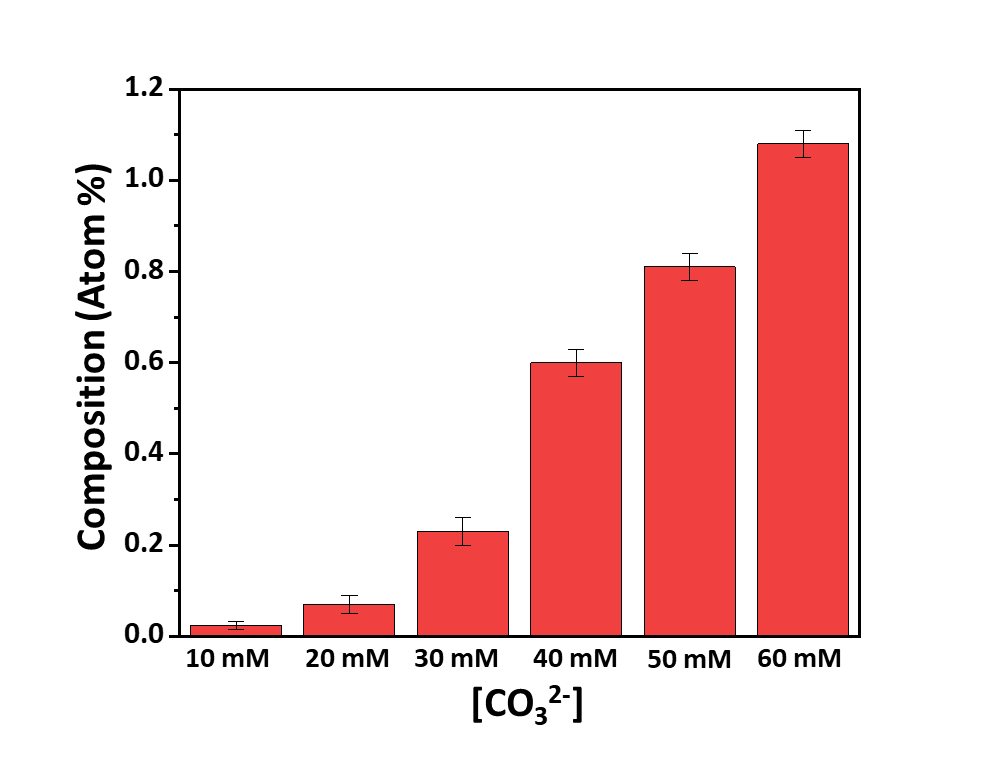
**

**Figure S13. The contribution of sodium (atom %) towards the composition of ACC spheres synthesized with varying [CO_3_^2-^].** This increasing trend with increasing [CO_3_^2-^] is suggestive of the fact that Na^+^ behaves as counter ions to the negatively charged CO_3_^2-^, making their way by diffusion, possibly allowing the maintenance of charge neutrality of the spheres.


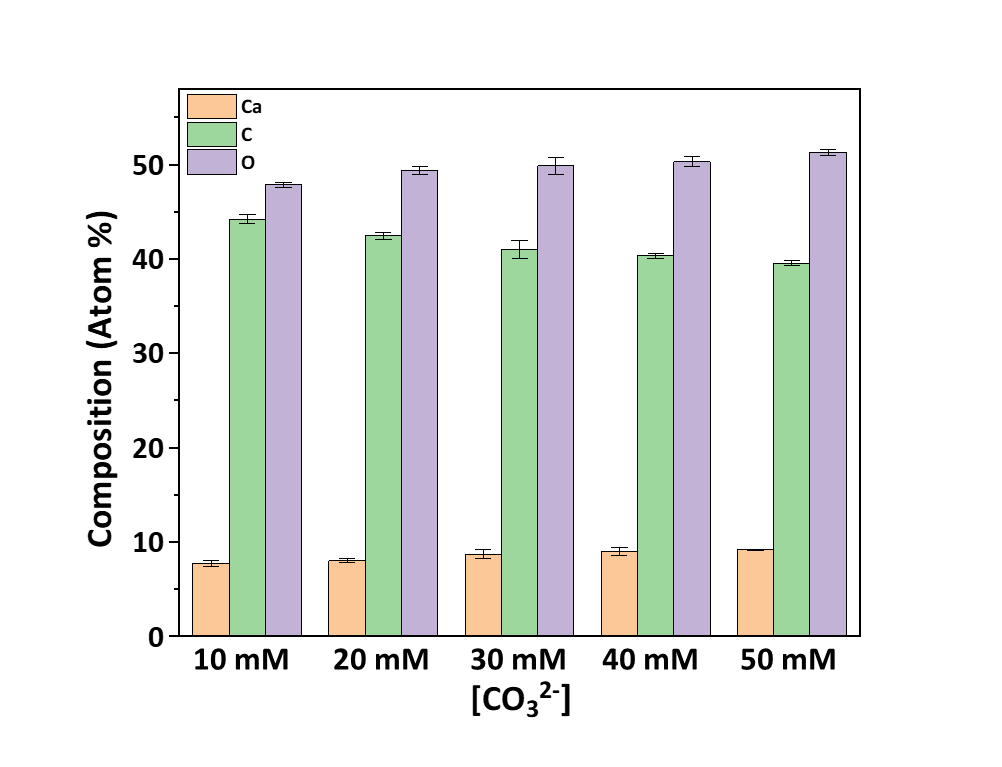


**Figure S14. Studying the kinetics of carbonate delivery into the PAA-Ca droplets using EDS.** The samples were obtained by sequential addition of CO_3_^2-^ at 10 mM increments (for example, the 50 mM sample here was made by adding 5 doses of 10 mM and letting the system equilibrate for 10 minutes in between). The elemental composition draws very close similarities to the ones where the addition was done instantly (represented in Fig. 3E).

**
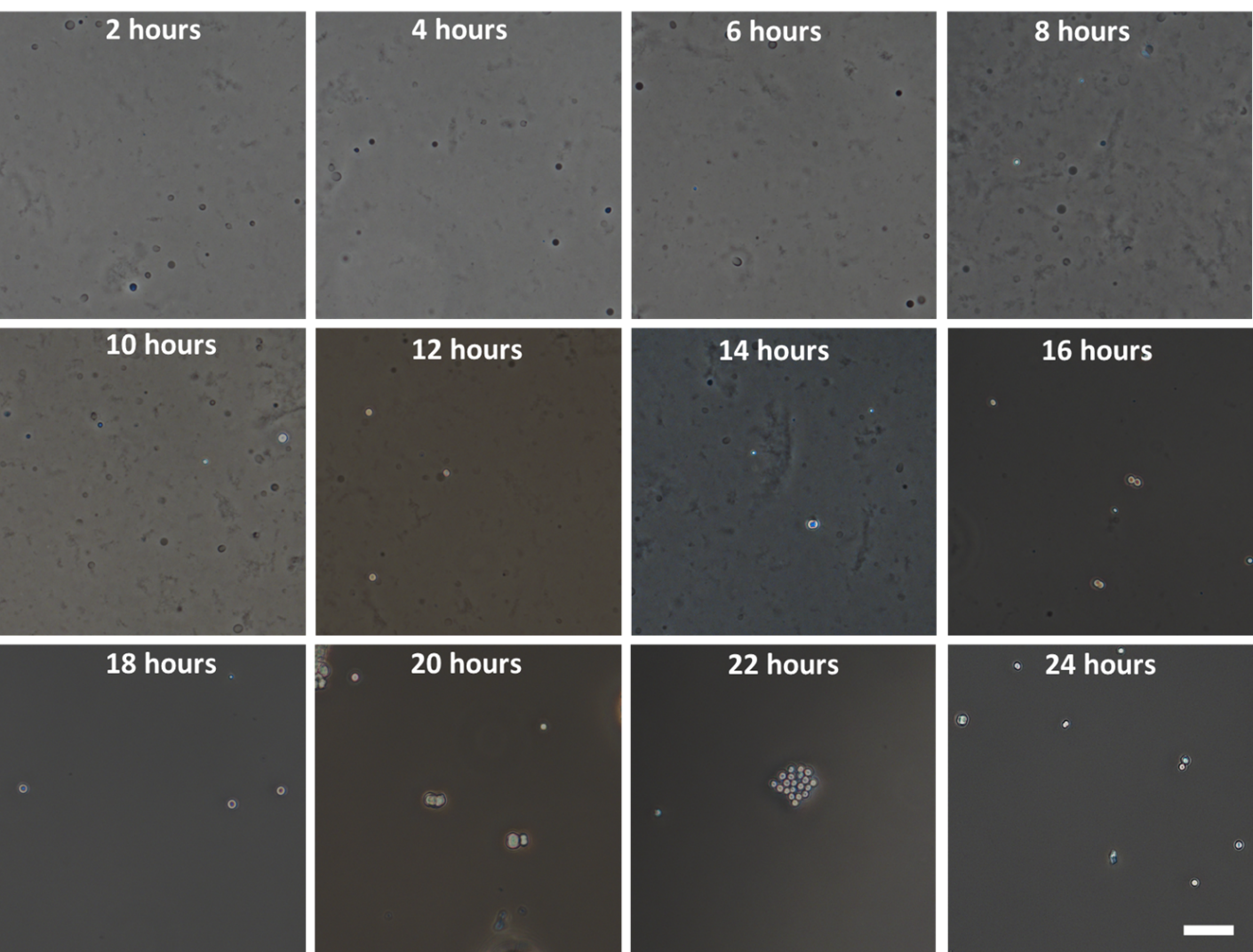
**

**Figure S15. Light microscope images at regular time intervals.** The crystallization of ACC with 60 mM CO_3_^2-^ was observed every two hours until complete crystallization after 24 hours. Here, we also observed that the ACC nanoparticles disappear after 14 hours, possibly due to dissolution. The sizes of the birefringent crystalline units are very similar to the ACC spheres observed during the early hours. The scale bar for all images is 20 μm.

**
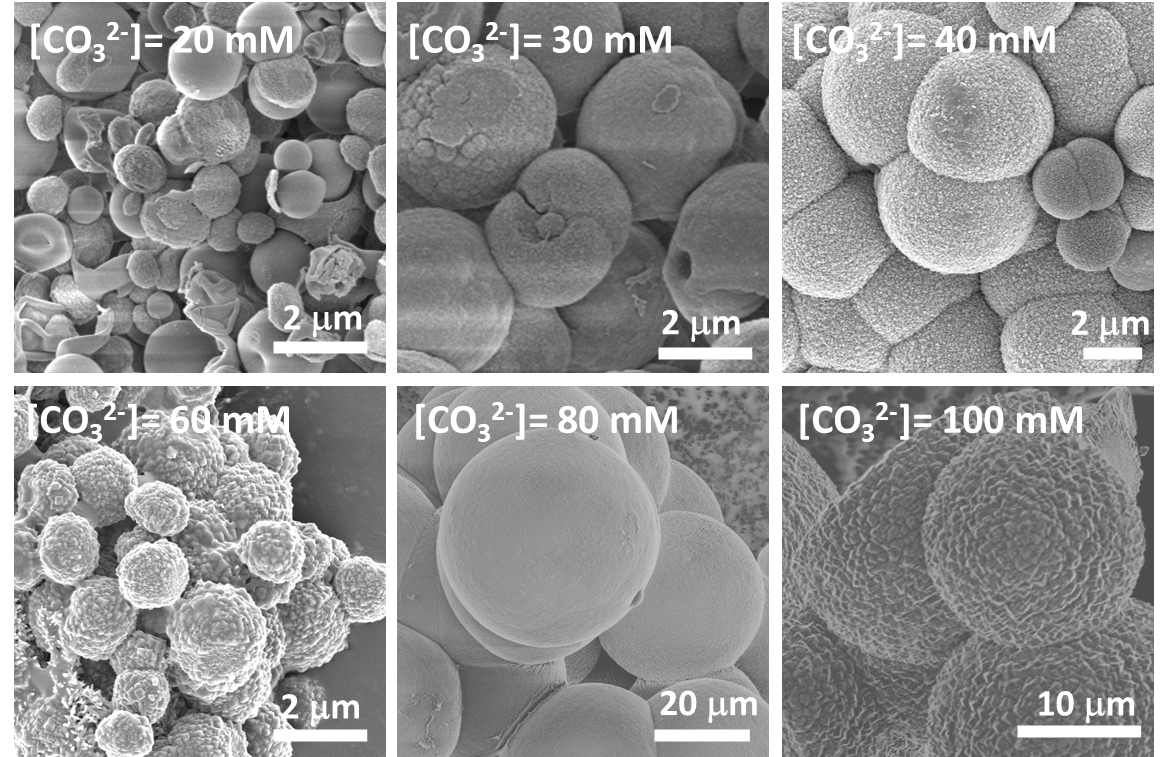
**

**Figure S16. SEM images of crystallized calcite synthesized with different [CO_3_^2-^].**
